# Is there a uniform approach to the management of diffuse parenchymal lung disease (DPLD) in the UK? A national benchmarking exercise

**DOI:** 10.1186/1471-2466-7-3

**Published:** 2007-03-13

**Authors:** Patricia Macedo, Robina K Coker, Martyn R Partridge

**Affiliations:** 1 Department of Respiratory Medicine, Hammersmith Hospitals NHS Trust, Ducane Road, London, W12 OHS, UK; 2 Department of Respiratory Medicine, NHLI Division, Imperial College London, Charing Cross Campus, St Dunstans Road, London, W6 8RP, UK

## Abstract

**Background:**

Benchmarking is the comparison of a process to the work or results of others. We conducted a national benchmarking exercise to determine how UK pulmonologists manage common clinical scenarios in diffuse parenchymal lung disease (DPLD), and to determine current use and availability of investigative resources. We compared management decisions to existing international guidelines.

**Methods:**

Consultant members of the British Thoracic Society were mailed a questionnaire seeking their views on the management of three common scenarios in DPLD. They were asked to choose from various management options for each case. Information was also obtained from the respondents on time served as a consultant, type of institution in which they worked and the availability of a local radiologist and histopathologist with an interest/expertise in thoracic medicine.

**Results:**

370 out of 689 consultants replied (54% response rate). There were many differences in the approach to the management of all three cases. Given a scenario of relapsing pulmonary sarcoidosis in a lady with multiple co-morbidities, half of respondents would institute treatment with a variety of immunosuppressants while a half would simply observe. 42% would refer a 57-year old lady with new onset DPLD for a surgical lung biopsy, while a similar number would not. 80% would have referred her for transplantation, but a fifth would not. 50% of consultants from district general hospitals would have opted for a surgical biopsy compared to 24% from cardiothoracic centres: this may reflect greater availability of a radiologist with special interest in thoracic imaging in cardiothoracic centres, obviating the need for tissue diagnosis. Faced with an elderly male with high resolution CT thorax (HRCT) evidence of usual interstitial pneumonia (UIP), three quarters would observe, while a quarter would start immunosuppressants. 11% would refer for a surgical biopsy. 14% of UK pulmonologists responding to the survey revealed they had no access to a radiologist with an interest in thoracic radiology.

**Conclusion:**

From our survey, it appears there is a lack of consensus in the management of DPLD. This may reflect lack of evidence, lack of resources or a failure to implement current guidelines.

## Background

Benchmarking is the comparison of a performance or process to the work or results of others. It involves learning, sharing information and adopting best practices to bring about improvements in performance. It was initially an exercise carried out in industry, but it is now widely used within healthcare. The process is so well established that the UK Government supports a public sector benchmarking service [1]. Benchmarking programmes in medical care have been set up to improve and maintain high standards of care for patients and to regulate and maintain training standards for healthcare professionals [2-4].

Diffuse parenchymal lung disease (DPLD) comprises over 200 conditions affecting the lung parenchyma. They are said to account for 15% of respiratory practice [5], but their clinical impact is probably underestimated [6-8]. The American Thoracic Society (ATS)/European Thoracic Society (ERS) revision of the classification of DPLD [9] standardised the classification of the idiopathic interstitial pneumonias (IIP), distinguishing between idiopathic pulmonary fibrosis (IPF) and IIPs other than IPF. Precise diagnosis in DPLD is important for prognosis and treatment. Establishing the diagnosis can be difficult and management options in many of these conditions are limited, often involving the use of immunosuppressants. The British Thoracic Society (BTS) published its first guidelines on DPLD in 1999 [10]. These addressed diagnosis and assessment of DPLD, and focused treatment recommendations on two major DPLDs, sarcoidosis and cryptogenic fibrosing alveolitis (CFA). The ATS published its guidelines on sarcoidosis in 1999[11] in conjunction in the European Respiratory Society (ERS) and the World Association of Sarcoidosis and Granulomatous Disorders (WASOG)[12], and for IPF in 2000 [13].

We carried out a benchmarking exercise to determine how pulmonologists in the United Kingdom (UK) manage common scenarios in DPLD. We wanted to see whether there was a consensus on the management of these cases and to review the use and availability of investigative resources. We compared the management chosen for three common scenarios to the recommendations in the ATS and BTS guidelines. To our knowledge, this is the first national benchmarking exercise for the management of DPLD in the UK.

## Methods

**Figure 1:**
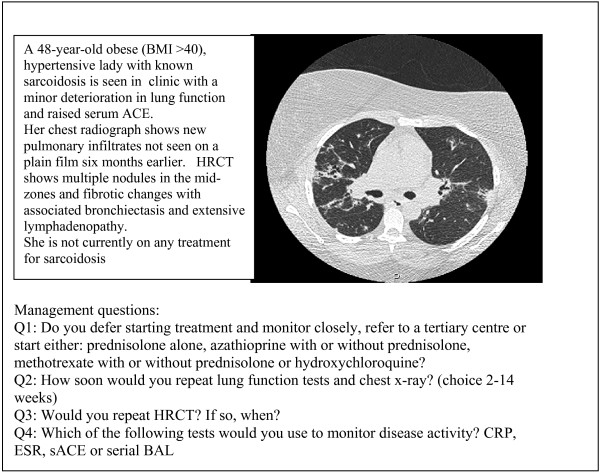
Case 1 – relapsing sarcoidosis. Abbreviations: HRCT: high resolution CT scan. CRP = C reactive protein; sACE = serum angiotensin converting enzyme. Serial BAL = serial bronchoalveolar lavage.

**Figure 2:**
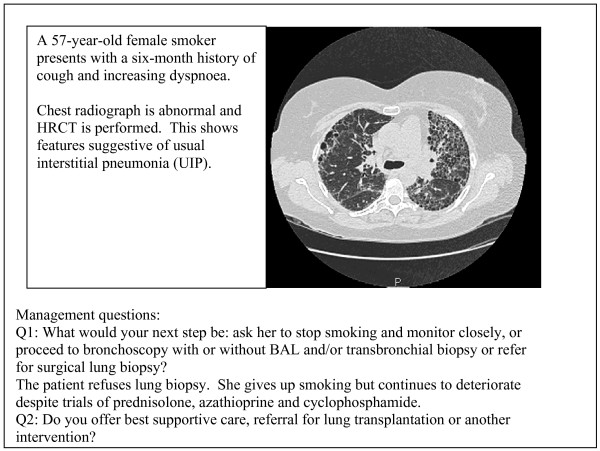
Case 2 – new onset DPLD. Abbreviations: BAL: bronchoalveolar lavage.

**Figure 3:**
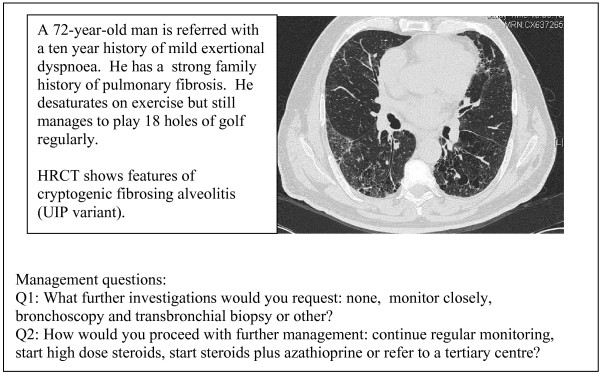
Case 3 – HRCT evidence of UIP in elderly male.

Six hundred and eighty nine consultant members of the British Thoracic Society were mailed a questionnaire seeking their views on three cases. Their opinion was sought on various management options. The cases chosen were common clinical scenarios in DPLD:

- relapsing sarcoidosis in a female with co-morbidities (case 1, figure 1)

- new onset DPLD in a 57 year old female (case 2, figure 2)

- an elderly male with HRCT evidence of usual interstitial pneumonia (UIP) (case 3, figure 3)

The clinical case scenarios were accompanied by an anonymous questionnaire requesting information regarding:

- length of time served as a consultant (0–5years, 5–10 years, 11–20years or >20years)

- type of institution in which they worked (District General Hospital, Associated University Hospital, Teaching Hospital or Cardiothoracic Centre)

- availability of local pathologist and radiologist with a declared interest and expertise in thoracic pathology/radiology

- whether or not they had sent out CT scans or histology slides for a second opinion in the last two years

The cases are summarised in figures 1, 2, 3. Each case scenario included one CT image, which had been reported by a radiologist with expertise in DPLD. Respondents were asked to choose from various management options suggested on the questionnaire. We looked at whether institution type or length of time served as consultant influenced management decisions.

The clinical case scenarios were fictional and the radiographic images anonymised. Ethics Committee approval was not required. No honorarium was provided for completing the questionnaire. The study was self funded.

## Results

**Figure 4:**
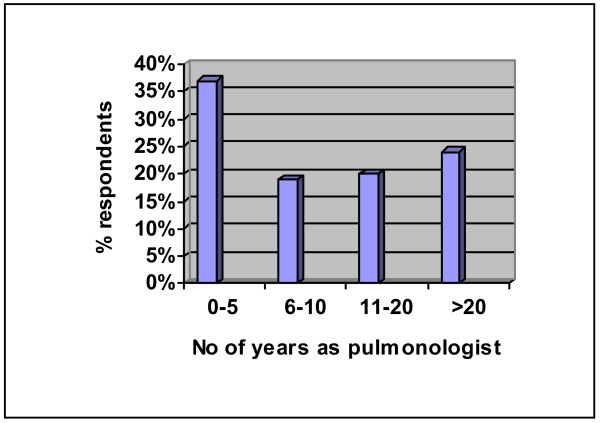
Length of time served as a Respiratory Specialist.

370 out of 689 physicians replied, giving a response rate of 54%. Just over a third of respondents (36%) had been a consultant for less than five years, with nearly a quarter having served for more than twenty years (figure 4).

**Figure 5:**
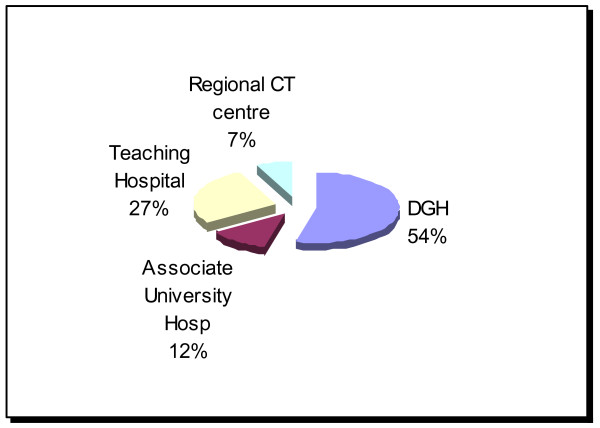
Respondents place of work. Abbreviations: DGH = provincial hospital.

Fifty four percent of consultants were based at district general hospitals (DGH) (secondary care centres), with 27% working at teaching hospitals (secondary and tertiary care centres) (figure 5). Eighty six percent of respondents said they had a radiologist at their hospital with a declared interest in thoracic radiology. Of the fourteen percent who did not, three quarters of these worked in DGHs. Just under fifty percent had sent out CT scans for a second opinion in the last two years. Only 48% said they had a pathologist at their institution with an expertise in thoracic pathology and in total, 53% had sent out histology specimens for a second opinion.

**Figure 6:**
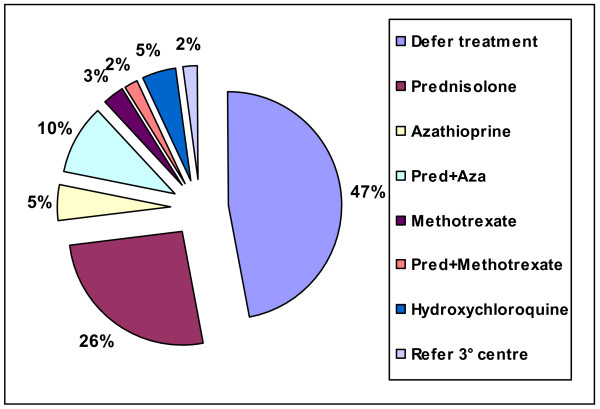
Treatment options chosen for case 1. Abbreviations: Pred = prednisolone; Aza = azathioprine.

When faced with a lady with relapsing sarcoidosis and multiple co-morbidities (case 1), 47% of respondents replied that they would defer starting treating and monitor. Of those who would institute therapy, prednisolone alone was the most popular choice (26%), with 10% choosing prednisolone plus azathioprine (figure 6).

When analyzing the data looking at the management decisions according to institution type or length of time served as consultant, there were some differences. Fifty percent of consultants from DGHs would defer starting treatment compared to thirty percent of consultants based at teaching hospitals. Similarly, one half of respondents who had been a consultant for less than five years would defer starting treatment compared to one third of those who had been a consultant for more than twenty years. There was no consensus on when to repeat lung function and chest radiograph but the majority suggested as interval of between 4–8 weeks.

Two thirds of all respondents said that they would not repeat a HRCT for case 1. Over a third of these consultants volunteered that the reason for not repeating the scan was that the waiting time at their institutions for a HRCT was very long.

For the final question in case 1, 13% of respondents said they would not use any of the recommended tests to monitor disease activity in sarcoidosis. Those who would use these tests, over fifty seven percent would use serum ACE, 22% CRP, 19% ESR and 2% would carry out serial bronchoalveolar lavage (BAL).

**Figure 7:**
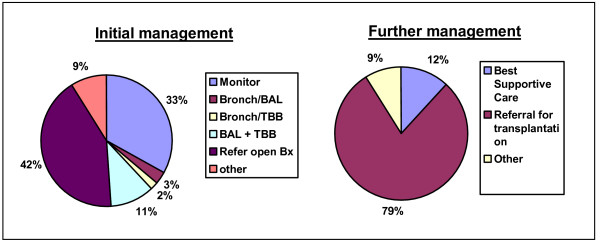
Replies for management options for Case 2. Abbreviations: Bronch – bronchoscopy; BAL – bronchoalveolar lavage; TBB – transbronchial biopsy; Bx – biopsy.

When faced with a fairly young lady with HRCT evidence of UIP (case 2), 42% of respondents would refer for open lung biopsy (figure 7). A third would simply monitor closely without any further invasive procedures. For the second question in case 2, after the patient continued to deteriorate, 79% of respondents would refer the lady for lung transplantation, with 12% offering best supportive care.

**Figure 8:**
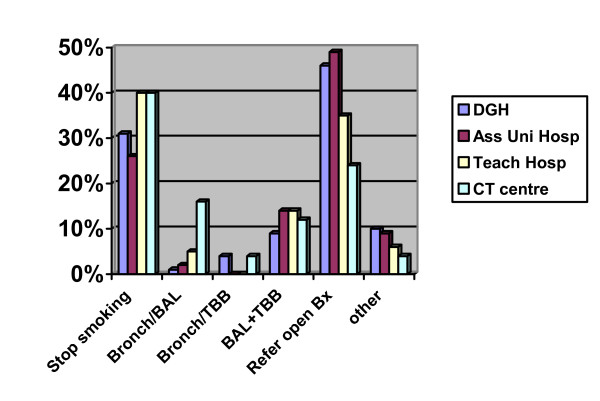
Replies according to Institution type – difference in referrals for surgical biopsies. Abbreviations: DGH = provincial (district general) hospital; Ass Uni Hosp = associated university hospital; Teach Hosp = teaching hospital; CT Centre = cardiothoracic centre; BAL = bronchoalveolar lavage; TBB = transbronchial biopsy; Bx = biopsy.

Looking at the impact of hospital type on management revealed that 50% of respondents from DGHs would refer for open lung biopsy compared to 24% of consultants from cardiothoracic centres (tertiary care) (figure 8).

**Figure 9:**
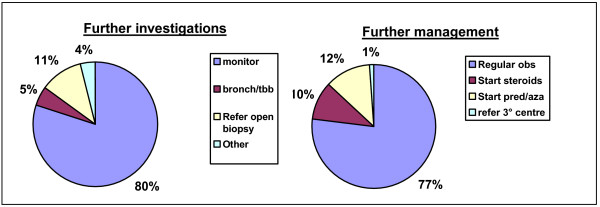
Replies for management options in Case 3. Abbreviations: Bronch = bronchoscopy; TBB = transbronchial biopsy; obs = observation; pred = prednisolone; aza = azathioprine.

In the final case (figure 9), the elderly man with HRCT evidence of UIP, 80% would monitor closely and not carry out any further investigations. 11% would refer for open lung biopsy, with 5% carrying out bronchoscopy with transbronchial biopsies. 77% of respondents would monitor the patient closely and would not commence immunosuppressant therapy. 10% would start high dose corticosteroids and 12% would start prednisolone plus azathioprine. There were no significant differences by institution type or time served as a consultant

## Discussion

Our study has revealed some significant differences amongst UK pulmonologists in their approach to managing three common scenarios in DPLD. In case 1, half of respondents would institute treatment with a variety of immunosuppressants while a half would simply observe. The BTS guidelines recommend prednisolone in relapsing pulmonary sarcoidosis and state that other anti-inflammatory agents should be reserved for patients who cannot take steroids, with chloroquine and methotrexate being the preferred agents. The ATS guidelines advocate the use of corticosteroids for patients with persistent pulmonary infiltrates and decline in spirometry, with antimalarials and cytotoxic agents for those requiring long term steroid therapy. Azathioprine was the preferred immunosuppressant after prednisolone in our survey. This may reflect the results of trials published since the guidelines [14,15]. Since the ATS/ERS/WASOG guidelines, many now regard azathioprine and methotrexate to be the recommended alternatives to prednisolone [16].

There was no consensus on when to repeat lung function or chest radiograph (the guidelines do not give recommended time intervals for these) for case 1. Interestingly, nearly fifty per cent of all respondents said they would use serum ACE to monitor disease activity. Serum ACE is elevated in approximately 60% of patients with sarcoidosis [17], but levels do not correlate with stage or prognosis. The BTS guidelines do not recommend routine measurement of serum ACE due to its poor sensitivity and specificity. In the ATS guidelines, serum ACE can be used as a marker of disease activity, but no opinion on its use is given, only that it is never diagnostic on its own. The ATS guidelines recommend following up patients with Stages II – IV at least three to six months, with physical examination, chest radiograph and lung function tests. The British guidelines to not give any advice as to when to follow up these patients, but it also recommends the use of chest radiographs and spirometry to monitor progress. Routine use of CT scanning is not recommended for sarcoidosis, but should be used in for patients with atypical clinical history or chest radiographs and to detect complications such as bronchiectasis and fibrosis.

Cases 2 and 3 dealt with two different patients with HRCT features suggestive of UIP. Both British and American guidelines state that in absence of an open lung biopsy, the diagnosis of IPF remains uncertain. The ATS guidelines [13] give major and minor criteria which can be applied in the absence of histology in order to make a diagnosis of IPF. Several studies have shown that experienced pulmonologists and radiologists can make a diagnosis of IPF on the basis on clinical and radiological data alone [18,19]. It is interesting to see that 42% of our respondents would have sent the lady in Case 2 for an open lung biopsy, despite her having a HRCT picture typical for UIP. Surgical biopsies should be reserved for when the diagnosis is uncertain [20] or there is an atypical clinical picture eg the patient is under 50 years old. Given the poor outcome in IPF, the ATS guidelines recommend treatment for all patients (unless there are contraindications) with corticosteroids, with or without immunosuppressive agents (azathioprine or cyclophosphamide). Treatment response should be reviewed at six months. The BTS guidelines suggest starting treatment if there has been a drop in lung function or progression of symptoms, with co-morbidities taken into account. It recommends starting treatment with prednisolone and azathioprine. Both set of guidelines recommend referral for transplantation for patients with progression of disease, and yet only eighty per cent of pulmonologists surveyed would have done so. In Case 3, only a quarter of respondents would have commenced immunosuppressants. Is this because they felt that in a 79 year old man the risks outweighed the benefits?

Many pulmonologists spontaneously reported difficulty in obtaining HRCT scans. HRCT scanning plays a key role in the diagnosis of DPLD. Its primary role is to distinguish between UIP and non-UIP. Certain forms of DPLD can be diagnosed on the basis of HRCT findings and clinical history, without the need for tissue diagnosis. Indeed, a recent North American survey showed that 67% of pulmonologists rely on HRCT to diagnose UIP without resorting to histological diagnosis [21]. In certain cases, it may assist in determining prognosis eg in IPF [22]. It can also help to determine the best site for surgical biopsy. Tissue diagnosis has been regarded by many as the gold standard diagnostic test in DPLD. Surgical biopsies may yield false negative results due to interlobar and intralobar variability [23]. The mortality rates related to open lung biopsies is quoted in the guidelines as than 1%. This figure may be an underestimation – Utz et al [24] reported a 17% 30-day surgical mortality in patients with UIP undergoing a surgical biopsy.

We appreciate there are several limitations in our study. The information provided in the case histories was limited, and the response rate was only 54%. However recent changes in the working practices of UK Respiratory Physicians has led to a significant degree of sub-specialisation and it's likely that those who did not respond are those who are majoring on the care of those with lung cancer or sleep apnoea, and rarely see patients with DPLD. Nevertheless, we feel the exercise has highlighted important issues. The study has revealed several "deviations" from existing guidelines. Studies in other fields have often suggested that physicians believe they adhere to guidelines but often don't [25]. Others have suggested that evidence based guidance is most likely to impact upon care if it's clear, relevant to practice, properly funded, supported by the profession and used in organizations that have established good systems for tracking guidance [26].

Do differences reflect poor implementation of guidelines' recommendations or lack of good evidence on which to make recommendations leading to diversity of management? Cabana M *et al *[27] have suggested that failure to implement guidelines can reflect (a) poor dissemination of guidelines (b) lack of belief in guidelines' recommendations (c) doubt about one's ability to deliver the guidelines' recommendations (d) reverting to previous practice. In this case it is possible that dissemination of the guidelines was less vigorous than for example asthma [28-30] and also possibly reflect that there has been a paucity of good trials in some of these areas, for example in the Cochrane Reviews on the value of oral steroids in pulmonary sarcoidosis [31] and IPF [32], only 12 trials could be used for pulmonary sarcoidosis. For IPF, all 15 studies which were identified had to be excluded due to inadequate methodologies. Lack of funding will also affect one's ability to research the questions necessary to fill gaps in evidence-based guidelines, and this is an especial problem in the field of DPLD [33].

Furthermore some may believe that the 1999 guidelines are now out of date and that their reported practice is more up to date [34]. However such an explanation cannot explain why one fifth of pulmonologists caring for a youngish person with terminal DPLD would not refer such a person for consideration of a lung transplant. The approach to the obtaining of confirmation of diagnosis by means of surgical biopsy also varied considerably between respondents. Many physicians have traditionally felt the need for absolute confirmation of diagnosis in the younger person in whom aggressive immunosuppression and eventual transplantation may be appropriate. This would explain the higher rate of referral for biopsy in Case 2 than Case 3. However it does not explain the paradox of more physicians in District General Hospitals reporting that they would refer a patient for a surgically obtained lung biopsy than would their opposite numbers in cardiothoracic centres where access to surgery was presumably easier. The paradox is most likely to reflect the greater likelihood of there being radiologists with a special expertise in thoracic radiology in cardiothoracic centres, and modern imaging techniques, coupled with radiological expertise which has permitted more accurate classification and differentiation of patients with DPLD – making a pathological diagnosis less necessary.

This non-availability of thoracic radiological expertise (and to a greater extent non-availability of lung pathology expertise) is a source of concern and suggests that patients may be suffering as a result and certainly appear to be more likely to have "unnecessary" surgical biopsies. This could be rectified either by a greater degree of radiological sub-specialisation or by the setting up of Regional Panels of Radiological and Pathological expertise in DPLD to whom images and biopsies could be referred [35,36]. The disparity between histological diagnosis made by general pathologists and specialist pathologists is highlighted in the study by Lettieri *et al *[37]. In this paper, histological diagnosis on interstitial lung disease biopsies made by general pathologists was different to that of a specialist pathologist in over 50% of cases, leading to changes in clinical management in a significant number of cases. Should DPLD be managed in designated centres in order to ensure a more uniform approach? Such an approach exists in other specialities. For example, in cystic fibrosis (CF), it is felt that patients are best cared for in CF specialist centres, offering an experienced multidisciplinary approach [38]. Often, there is shared care with the local hospital. This allows the patient to be seen locally for convenience, whilst at the same time, being able to make use of the facilities and resources of the specialist centre. In any case, it is clear that resources need to be made more available throughout the country.

Professional judgement by individual doctors is now being challenged by the need to be shown to be producing a cost effective service with uniform outcomes. We would suggest that whilst benchmarking or similar such processes have previously been used mainly by healthcare managers to look at hospitalisation rates and length of hospital stay, it can provide an insight into other differences in process of care which may both stimulate new research questions and also service production and implementation of guidelines.

## Conclusion

From our benchmarking exercise, it appears that in the UK there is a lack of consensus in the management of DPLD. This may reflect lack of evidence, lack of resources or a failure to implement existing guidelines.

## Competing interests

The author(s) declare that they have no competing interests.

## Authors' contributions

MRP and RC had the original idea for the study, PM analysed the results, PM, MRP and RC wrote the paper and MRP is the paper's guarantor.

All authors have read and approved the final manuscript.

## Pre-publication history

The pre-publication history for this paper can be accessed here:


